# A Linearization Time-Domain CMOS Smart Temperature Sensor Using a Curvature Compensation Oscillator

**DOI:** 10.3390/s130911439

**Published:** 2013-08-28

**Authors:** Chun-Chi Chen, Hao-Wen Chen

**Affiliations:** Department of Electronic Engineering, National Kaohsiung First University of Science and Technology, Kaohsiung, 811, Taiwan; E-Mail: u9752018@nkfust.edu.tw

**Keywords:** curvature compensation, smart temperature sensor, oscillator, time-domain, delay line

## Abstract

This paper presents an area-efficient time-domain CMOS smart temperature sensor using a curvature compensation oscillator for linearity enhancement with a −40 to 120 °C temperature range operability. The inverter-based smart temperature sensors can substantially reduce the cost and circuit complexity of integrated temperature sensors. However, a large curvature exists on the temperature-to-time transfer curve of the inverter-based delay line and results in poor linearity of the sensor output. For cost reduction and error improvement, a temperature-to-pulse generator composed of a ring oscillator and a time amplifier was used to generate a thermal sensing pulse with a sufficient width proportional to the absolute temperature (PTAT). Then, a simple but effective on-chip curvature compensation oscillator is proposed to simultaneously count and compensate the PTAT pulse with curvature for linearization. With such a simple structure, the proposed sensor possesses an extremely small area of 0.07 mm^2^ in a TSMC 0.35-μm CMOS 2P4M digital process. By using an oscillator-based scheme design, the proposed sensor achieves a fine resolution of 0.045 °C without significantly increasing the circuit area. With the curvature compensation, the inaccuracy of −1.2 to 0.2 °C is achieved in an operation range of −40 to 120 °C after two-point calibration for 14 packaged chips. The power consumption is measured as 23 μW at a sample rate of 10 samples/s.

## 1. Introduction

Temperature sensors are commonly used for temperature sensing and are widely applied in measurements, instrumentation, and control systems. With the tremendous growth of the industry, the requirements of most modern automatic control systems cannot be fulfilled by using resistance temperature detectors (RTDs), thermistors, or thermocouples. By contrast, semiconductor temperature sensors offer many advantages reflecting the changing trends of the mainstream market. They not only provide better noise immunity through a higher-level output signal, but also generate logic outputs that can communicate directly with digital systems, thus playing an important role in thermal or power management systems. Based on the demand for small size and high performance, integrated temperature sensors made with CMOS technology are more competitive and attractive when compared to others of their type.

For further integration with digital VLSI systems and to reduce the tasks of the microcontroller, analogue-to-digital converters (ADCs) were gradually integrated into thermal sensors to compose the so-called “intelligent or smart temperature sensors” [1–4]. The test temperature is usually converted first into a voltage signal by a temperature sensor, and then a corresponding ADC is used for subsequent digital output coding. Usually, an ADC with more than 10 output bits is required to obtain the necessary resolution at the expense of large chip area and power consumption. Furthermore, to be fully compatible with the standard digital CMOS technology and reduce measurement errors, sophisticated circuits accompanied by additional on-chip elaborated calibration techniques that increase the circuit complexity and consume more chip area and power are generally adopted.

**Figure 1.**
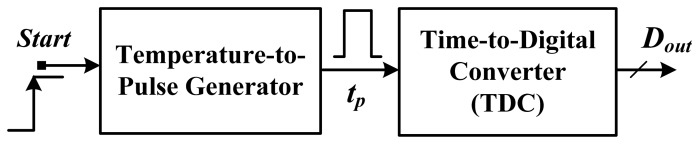
Block diagram of the time-domain smart temperature sensor [5].

In recent years, an innovative time-domain smart temperature sensor was proposed to substantially reduce the cost and circuit complexity of smart sensors for VLSI systems, as shown in Figure 1 [5]. The inverter-based temperature-to-pulse generator as a temperature sensor was used to convert the test temperature into a pulse with a width PTAT. The generated pulse was then fed into a time-to-digital converter (TDC) rather than an ADC to produce the corresponding digital output. In a TSMC 0.35-μm CMOS digital process, the chip size was only 0.09 mm^2^, and the measured error was ±0.6 °C after two-point calibration within a −40 to 60 °C temperature range. The power consumption is 9 μW@5 samples/s. For facile VLSI or system-on-chip (SOC) integration, a fully digital smart temperature sensor realizable with field programmable gate array (FPGA) logic was proposed [6]. The FPGA chips were measured to have an error of −1.5–0.8 °C after two-point calibration over a temperature range of 0–75 °C. With two current-starved oscillators controlled by reference current and PTAT current respectively, the temperature sensor in a 0.18-μm CMOS process has an area of 0.05 mm^2^ and an inaccuracy of −1.6–3.0 °C within a 0–100 °C range after two-point calibration. It consumed only 220 nW@100 samples/s [7]. Based on a linear MOS operation, another ultra-low power of 405 nW@1 k samples/s CMOS smart sensor with a differential temperature sensing delay generator was proposed [8]. The chip size is 0.03 mm^2^ in a 0.18-μm CMOS, and the inaccuracy is −0.8–1.0 °C from 0 °C to 100 °C after two-point calibration. Furthermore, to reduce the high-volume production cost, an all-digital inverter-based temperature sensor was proposed to perform self-calibration by using a reference clock to remove the effect of process variation for one-point calibration [9]. The inaccuracy of −5.1–3.4 °C for three test chips was measured in the range of 0–60 °C. The core area is 0.01 mm^2^ in a 65-nm CMOS, and the power consumption is 150 μW@10 k samples/s. Later, another FPGA-based version for one-point calibration support and circuit size reduction was proposed [10]. By adopting an additional off-chip second order master curve for curvature correction, the measurement errors for 20 test chips were successful reduced to −0.7–0.6 °C over 0–100 °C range. Only 48 logic elements were realized, and 175 μW@1 k samples/s was consumed. Also with one-point calibration, a frequency-to-digital-converter-based smart sensor that involves using a multiphase clock was proposed to achieve an inaccuracy of −2.7–2.9 °C within a range of −40 °C to 110 °C [11]. The circuit featured the fastest conversion rate of 366 kHz and a small size of 0.0066 mm^2^ in a 65-nm CMOS technology.

**Figure 2.**
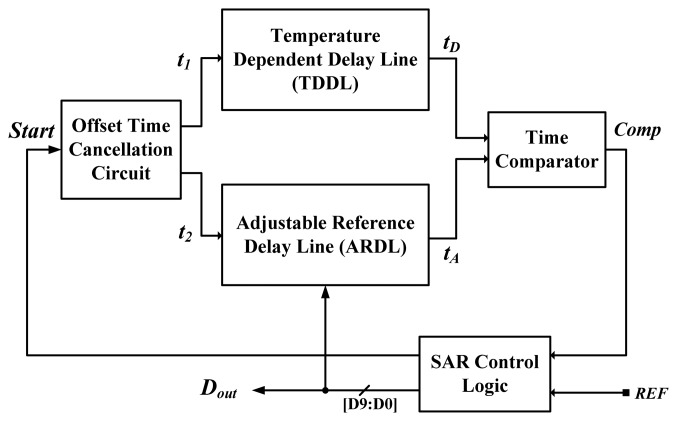
Block diagram of former smart temperature sensor with curvature compensation [12].

Compared with voltage-domain sensors [1–4], time-domain inverter-based sensors possess the advantages of low cost and simple structure, but exhibit the disadvantage of poorer linearity because a large curvature exists on the temperature-to-delay transfer curves of the inverter-based delay line. To enhance linearity, the on-chip curvature compensation technique between two linear delay lines was invented to achieve the highest accuracy among the related time-domain sensors [12]. The architecture of the successive approximation algorithm (SAR) smart temperature sensor is redrawn in Figure 2. For temperature sensing, the temperature-dependent delay line (TDDL), similar to that in [5], is used to generate a PTAT delay. An offset time cancellation circuit composed of two D-type flip-flops (DFFs) replacing a large number of offset-compensation delay cells, was used to reduce the time offset faced at the lower test temperature bound. The SAR control logic, adjustable reference delay line (ARDL) and time comparator can be viewed as an equivalent TDC to convert the TDDL delay into the corresponding digital output. Consequently, more chip area was consumed. With a 10-bit output design, the size in the 0.35-μm CMOS was increased to 0.6 mm^2^, which is much larger than the original versions [5]. The curvature of the ARDL curve was designed to compensate that of TDDL curve for curvature compensated operation and its inaccuracy of −0.25–0.35 °C is the best in the operation range of 0–90 °C. However, the error is not decreased significantly for a wider operation range of −40 to 120 °C by using its curvature compensation technique [12]. Additionally, because this kind of sensor [5–12] is much more sensitive to the supply voltage quality [13], the inaccuracy with voltage variation is rather poor. The supply voltage can be regulated to reduce the voltage sensitivity to stabilize the performance of the sensors when necessary.

To keep the main advantages of time-domain sensors with simple structure for low-cost VLSI systems, a cost-efficient CMOS smart temperature sensor is presented. To enhance accuracy and extend the operation temperature range, a simple but effective on-chip curvature compensation oscillator is adopted for linearization of the sensor output. The rest of the paper is organized as follows: the characteristic curvature of the transfer curve of the inverter-based sensor is described in Section 2. Section 3 introduces the circuit of the proposed sensor and the curvature compensation. The experimental results are illustrated and discussed in Section 4. Finally, Section 5 provides the conclusions of this study.

## 2. Curvature Characteristic of Inverter-Based Smart Temperature Sensors

**Figure 3.**
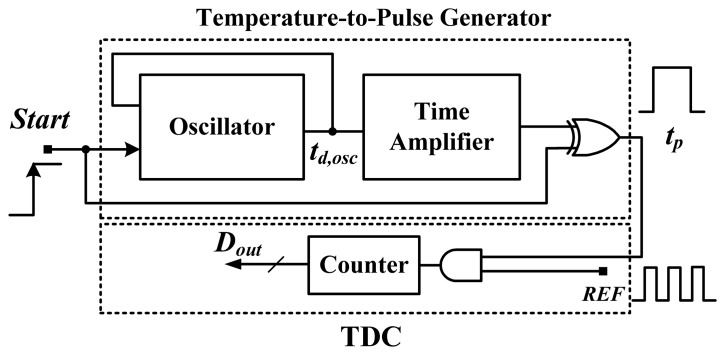
Block diagram of a smart temperature sensor with a reference clock adapted from [6].

The block diagram of the smart temperature sensor with a reference clock is shown in Figure 3 [6]. Its structure may be simplest among the various proposed time-domain smart temperature sensors. An oscillator composed of a NAND gate and an inverter-based delay chain is used to generate the period width *t*_*d*,*osc*_.

For adequate resolution and cost reduction, the time amplifier realizable with a circulation counter and a comparator is used to amplify the oscillatory period according to a preset circulation number [6]. The succeeding XOR gate is then used to obtain an adequately wide output pulse *t_p_*. Finally, an off-chip reference clock is used to count the *t_p_* width with an output counter for time-to-digital conversion. The reference clock and the output counter can be regarded as the simplest TDC. The period width *t*_*d*,*osc*_ can be written as in Equation (1):

(1) $$t_{d,\text{osc}}=2\cdot k\cdot t_{d}$$

where *k* and *t_d_* are the number of gates (including the NOT and NAND gates) in the cyclic delay line and the average propagation delay of those gates, respectively. The delay signal *t_d_* is a thermally sensitive quality that can be used to sense the test temperature [5,6,9–12], and its propagation delay with temperature dependence is approximated as [14]:

(2) $$t_{d}\;(T)=\frac{2LC_{L}}{WC_{\text{OX}}V_{\text{DD}}}\times \frac{1}{\mu (T)}\times \frac{\ln(3-4V_{T}\;(T)/V_{\text{DD}})}{1-V_{T}\;(T)/V_{\text{DD}}}$$

where T, W/L and C_L_ are the operation temperature, effective aspect ratio of the transistors, and the loading capacitance of the NOT gates. The temperature dependencies of mobility and threshold voltage have been formulated to be [15]:

(3) $$\mu (T)=\mu_{0}\times {\left(\frac{T}{T_{0}}\right)}^{\text{km}},\text{km}=-1.2\sim -2.0$$

(4) $$V_{T}(T)=V_{T}(T_{0})+\alpha (T-T_{0}),\alpha =-0.5\sim -3\text{mV}/{}^{\circ}C$$

in which *T_0_* is the reference temperature. Because *α* is extremely small and *V_T_ (T)* is further divided by V_DD_ in Equation (2) to reduce its impact on *t_d_ (T)* (*V_DD_* ≫ *V_T_*(*T*) in this case), the thermal effect of *V_T_* is ignored for simplicity and the thermal effect of *t_d_(T)* is dominated by the mobility. Therefore, the thermal coefficient of *t_d_(T)* is positive:

(5) $$\begin{array}{l} t_{d,\text{osc}}(T)=2\cdot \text{k}\cdot t_{d}(T)\\ =2\cdot \text{k}\cdot \frac{2LC_{L}T_{0}^{\text{km}}}{\mu_{0}WC_{\text{OX}}V_{\text{DD}}}\times \frac{\ln(3-4V_{T}/V_{\text{DD}})}{1-V_{T}/V_{\text{DD}}}\times \frac{1}{T^{\text{km}}}=\gamma \times T^{-\text{km}} \end{array}$$

where γ is a process-sensitivity constant and is nearly temperature-independent, and the exponent −*km* of temperature T considered to be independent of temperature [16] dominates the thermally dependent term. If *km* = −1 ideally in Equation (5), the period width proportional to the temperature is perfectly linearity. However, the parameter *km* is controlled by doping level [17] and its value expands within a range of −1.2–−2, meaning that a concave curvature always exists on the transfer curve of the thermal delay line. Therefore, the cyclic delay line can act as a time-domain PTAT temperature sensor that has a much simpler structure than that of the conventional voltage-domain sensor at the expense of poorer linearity.

To improve resolution, the thermal period width *t*_*d*,*osc*_*(T)* is amplified to generate an adequately wider delay by using the time amplifier with a preset value *n*. With the XOR gate, the mask signal *t_p_* is generated and can be simply calculated as *t_p_(T)* = *n* · *t*_*d*,*osc*_*(T)*. The delay of the time amplifier is ignored because its amount is greatly smaller than the amount of *n* · *t*_*d*,*osc*_. Width *t_p_* is then counted with a reference clock *t_REF_* to obtain the following output code *D_out_*. Hence, *D_out_* can be estimated as:

(6) $$D_{\text{out}}(T)=\frac{t_{p}(T)}{t_{\text{REF}}}=\frac{n\cdot t_{d,\text{osc}}(T)}{t_{\text{REF}}}=\frac{n\cdot \gamma }{t_{\text{REF}}}\times T^{-\text{km}}$$

Because the value *n* and the reference clock *t_REF_* are constant and ideally temperature-insensitive, the linearity of the sensor digital output inevitably resembles that of the thermal oscillator. As mentioned, because a curvature exists on the oscillator output curve, the curve of the sensor output will show the same curvature. Based on the simple structure shown in Figure 3, a small area, easy design, but poor linearity can be expected.

**Figure 4.**
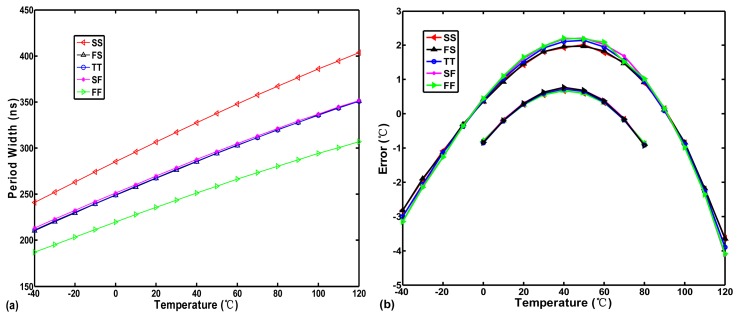
(**a**) Corner analysis and (**b**) Corresponding inaccuracies of the inverter-based oscillator.

To further determine the effect of the exponent of T versus the temperature error, the temperature-to-time (period width) transfer curve of the inverter-based oscillator (one NAND and ten NOT gates in series) in Figure 3 was simulated with five process corner variations in a 0.35-μm TSMC CMOS digital process, as illustrated in Figure 4a.

The curves of *t*_*d*,*osc*_(*T*) with some curvature are slightly convex. The corresponding inaccuracies of −4.1–2.1 °C within a range of −40–120 °C and of −0.9–0.7 °C within a range of 0–80 °C both generated from the transfer curves in Figure 4a are shown in Figure 4b. Because the parameter *km* varies slowly with the doping level, it can be regarded as a constant with process variation for a given fabrication process [10]. Consequently, the error curves with five process corner present nearly the same curvature. Obviously, according to Equation (5), the convex curve does not match the value of −*km* of T, 1.2–2.0. This means that, although the mobility dominates the term T, the threshold voltage also affects it slightly to make the transfer curves show a convex curvature rather than a concave curvature. Therefore, Equation (5) and Equation (6) can be simply modified as:

(7) $$t_{d,\text{osc}}(T)=\gamma \times T^{-km^{'}}$$

(8) $$D_{\text{out}}(T)=\frac{n\cdot \gamma }{t_{\text{REF}}}\times T^{-km^{'}}$$

where −*km′* is the actual curvature order of the transfer curve in a given process. The value of −*km′* should be less than 1 because of the convex curve of *t*_*d*,*osc*_(*T*) shown in Figure 4a. Moreover, as illustrated in Figure 4b, the wider the temperature range is, the higher the non-linear error becomes because of the curvature. The error for −40–120 °C is nearly four times that for 0–80 °C. The larger the deviation value between −*km′* and 1, the larger the curvature of the transfer curve occurs and the corresponding accuracy of the sensor is also lower. Thus, the curvature compensation for the thermal transfer curve must be performed to make the sensors suitable to operate with the wider temperature range as −40–120 °C. As long as the value of exponent of T on sensor output can be corrected to be 1 as closely as possible, the inaccuracy can be decreased greatly.

## 3. Circuit Description and Curvature Compensation

**Figure 5.**
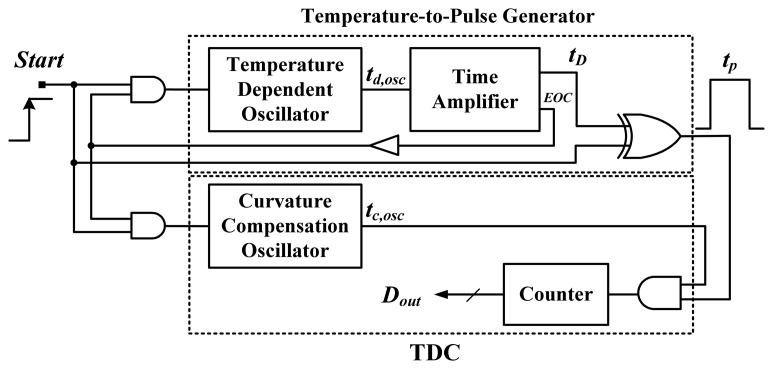
Block diagram of the proposed smart temperature sensor.

**Figure 6.**
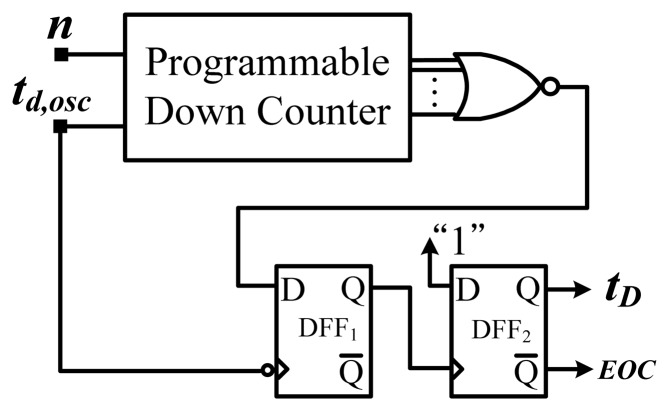
Structure of adopted time amplifier.

Figure 5 shows the block diagram of the proposed smart temperature sensor with an on-chip curvature compensation oscillator. The circuit of the temperature-to-pulse generator is composed of a retriggerable ring oscillator (one NAND and sixteen NOT gates), a time amplifier, and an XOR gate. The generator is used to generate pulse *t_p_* with a PTAT width. Furthermore, an end-of-conversion (EOC) signal is issued to shut down the two oscillators to save power, with the help of two AND gates. To ensure correct counts, a delay buffer is inserted to delay the EOC. The time amplifier, shown in Figure 6, is used for time amplification to obtain a sufficient temperature resolution. A fixed value of n = 1,024 (amplification factor) in the time amplifier was adopted to amplify the period width. The additional DFF_1_ was inserted for deglitching. A larger preset input value results in higher output circulation times of the oscillatory period and a longer thermal delay *t_D_*.

As mentioned, the *t*_*d*,*osc*_ generated by the inverter-based oscillator shows a curvature, and thus the corresponding errors increase non-linearly as the temperature ranges increase. To enhance the accuracy and broaden the temperature operation range, the on-chip curvature compensation oscillator is adopted to compensate and simultaneously quantize the PTAT pulse *t_p_* into a linearization digital output through the help of the output counter. Compared with the former smart sensor [12], the proposed sensor is designed to lessen both unreasonable delay line length requirements by replacing the linear delay-line-based structure with the oscillators-based structure. Without the use of a large number of the logic gates, the temperature resolution can be improved without significantly increasing the chip area. Furthermore, the SAR control logic, time comparator, and offset time cancelation circuit in [12] are removed. The proposed structure is much simpler than that of the former version. This greatly reduces the circuit complexities, and the chip area reduction can be expected.

By using the curvature compensation oscillator to replace the reference clock, and assuming that the sensor output is ideally linear after the hypothetically perfect compensation, the exponent of T on the sensor output should be predicted as 1. In this situation, we have:

(9) $$D_{\text{out}}(T)=\frac{n\cdot t_{d,\text{osc}}(T)}{t_{c,\text{osc}}(T)}=\beta T^{1}$$

where *t*_*c*,*osc*_*(T)* is the period width of the compensated oscillator with temperature dependence and β is another proportional constant nearly independent of temperature. By using Equation (7) and (9), the temperature dependence of the desired oscillator can be reversely evaluated as:

(10) $$t_{c,\text{osc}}(T)=\frac{n\cdot \gamma }{\beta }\times T^{-km^{'}-1}$$

This roughly provides the desired curve characteristic of the oscillator for linearization. If the value of *km′* is determined in a given process, then the temperature dependence of the curvature compensation oscillator can be predictable by using (10). As previously mentioned, −*km′* is less than 1 in the TSMC 0.35-μm CMOS process, and the exponent of T on *t*_*c*,*osc*_*(T)* would be less than 0 for linearity improvement, meaning that the pulse width of the desired oscillator should be slightly decreased with increasing temperature. In [12], the curve characteristic on the compensation delay line was slightly increased with increasing temperature, which does not conform to the curve characteristic as Equation (10), and the simulated error of the sensor is as high as −2.0–2.5 °C for a −40 to 120 °C range.

**Figure 7.**
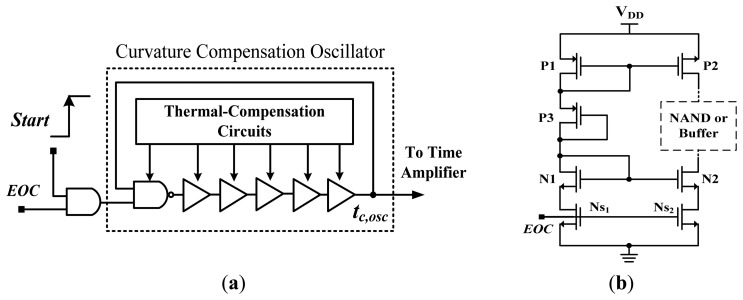
Schematics of (**a**) Curvature compensation oscillator and (**b**) Adopted thermal-compensation circuit.

The proposed curvature compensation oscillator consists of a NAND gate and a buffer-based delay chain (ten NOT gates), both under thermal-compensation conditions for curvature compensation, as shown in Figure 7a. The thermal-compensation circuit also adopted in [12] was used to reduce the thermal-sensitivity of the buffer (or NAND gate) in the oscillator, as shown in Figure 7b. The diode-connected transistors P1, N1, and P3 serve as the core of the thermal compensation circuit. The P1, P3, and N1 will operate in the saturation region because they are all diode connected. If an optimal bias voltage is designed for transistor P3 as:

(11) $$V_{\text{GS},P3}=V_{T}(T_{0})+\alpha (T-T_{0})+2\frac{\alpha \cdot T}{\text{km}}$$

then the corresponding conduction current of the bias network is proven to be [5]:

(12) $$I_{D,P3}=\frac{1}{2}\mu_{0}C_{\text{OX}}\left(\frac{M}{L}\right){\left(\frac{T}{T_{0}}\right)}^{\text{km}}{\left[\frac{2\alpha T}{\text{km}}\right]}^{2}(1+\lambda V_{\text{GS},P3})$$

**Figure 8.**
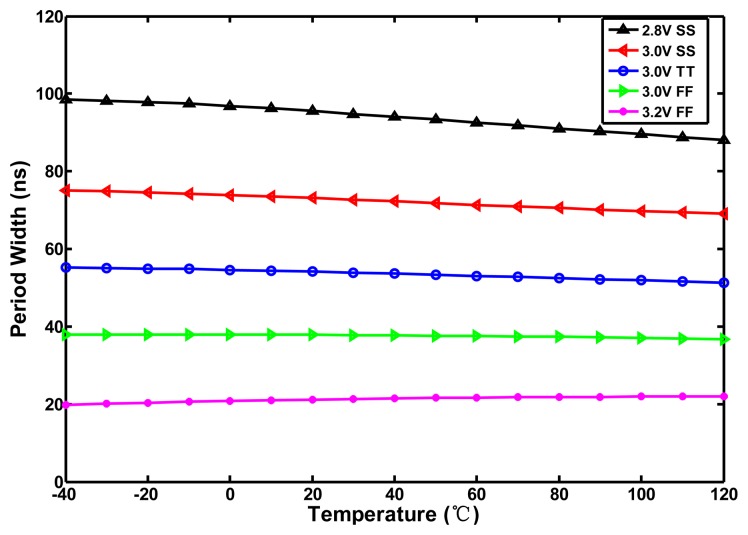
Simulation results of curvature compensation oscillator.

**Figure 9.**
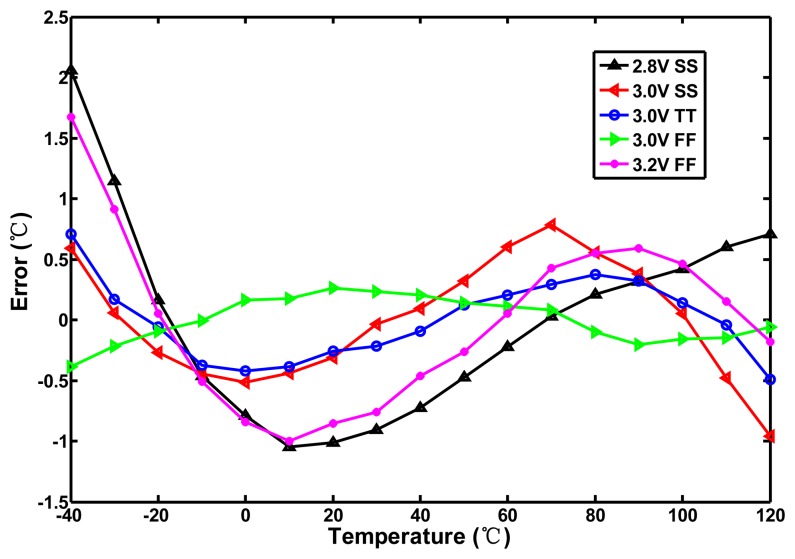
Simulated errors of proposed sensor for −40–120 °C.

When *km* equals −2, the conduction current becomes completely temperature-independent. Although the actual value of *km* expands within a range of −1.2–−2.0, the temperature dependence of the delay element is still reduced greatly. Using Equation (11) and (12) one can provide the first cut design to generate the period width of the oscillator with low thermal sensitivity. For simplification of the circuit design, the proposed curvature compensation circuit is developed based on the compensation circuit in [12]. With the help of HSPICE, the size of the thermal-compensation transistors P3, P1, and N1 can be further adjusted properly to match the curvature of the desired curve to be as close to that predicted by Equation (10) as possible. To save power again, the operation of the compensated circuit can be turned off by the EOC. With HSPICE simulation, Figure 8 shows the temperature-to-time transfer curves of the proposed oscillator with curvature compensation. With a supply voltage of 3.0 V and process corners (3.0 V SS, 3.0 V TT, 3.0 V FF), the thermal-compensated widths are decreased as temperature increases, which conforms to the desired characteristic as stated previously. The corresponding INL errors of the proposed circuit are simulated in Figure 9. With the curvature compensation under the voltage of 3.0 V, the error is successfully reduced from −4.1–2.1 °C to −1.0–0.8 °C better than −2.0–2.5 °C in [12] for −40–120 °C range. The linearity was greatly improved with the utilization of the proposed oscillator. With only proper sizing of the compensation transistors in the compensated circuit, the curve characteristic on the compensated oscillator was determined and the significant inaccuracy improvement can be achieved with only slightly increasing circuit complexity and area. However, with the simulation results under the voltage and process variations, the worst inaccuracy (occurring in 2.8 V SS) is −1.1–2.1 °C because the curvature compensation circuit does not perform well.

## 4. Measurement Results

**Figure 10.**
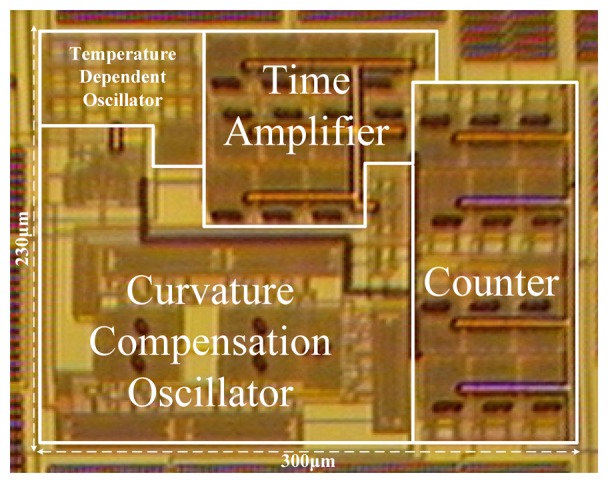
Microphotograph of the proposed sensor.

Figure 10 shows a microphotograph of the proposed sensor with a chip area of merely 0.07 mm^2^ in a TSMC 0.35-μm CMOS process. Compared with the former version [12], this achieved a nearly ten-fold improvement in chip size. With such a simple structure, the chip size of the proposed circuit is the smallest among the related inverter-based sensors when taking CMOS process into consideration [5–12].

**Figure 11.**
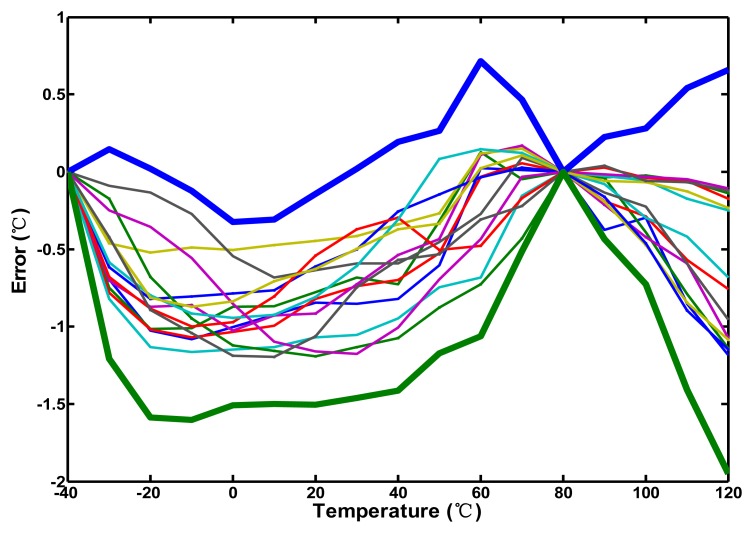
Measurement errors in the range of −40–120 °C for 14 test chips; bold lines indicate the ±3 σ values.

To demonstrate the performance of curvature compensation, the measurements were performed in 10 °C steps from −40 °C to 120 °C in the programmable temperature and humidity chamber MHG-120AF which was recalibrated by PT-100 before the measurement to figure out the actual performance of the test chips. The FPGA control board issued the test input signal, and a logic analyzer collected the digital output codes. An Agilent DSO7054A digital oscilloscope was also used to verify the timing of the measurement system. Due to the curvature compensation with 3.0 V supply voltage, the measurement errors for 14 test chips were within −1.2–0.2 °C after two-point calibration and the corresponding 3 σ error is −1.9–0.7 °C, as shown in Figure 11. Two-point calibration was fulfilled by performing linear curve fitting with the digital outputs of −40 °C and 80 °C, which were chosen to minimize error. A nearly five-fold improvement in accuracy is achieved when compared with the simulation inaccuracy without curvature compensation. This demonstrates that the proposed curvature compensation technique for linearity enhancement functions.

**Figure 12.**
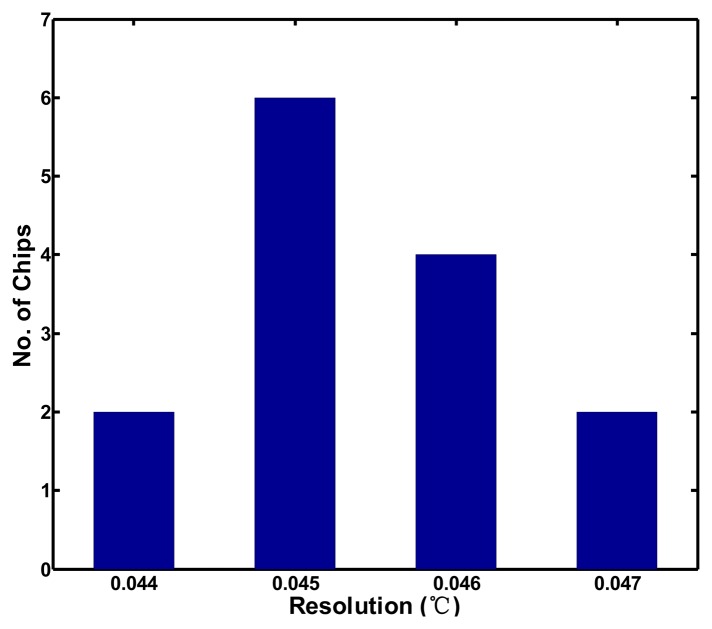
Resolution histogram of 14 test chips.

**Figure 13.**
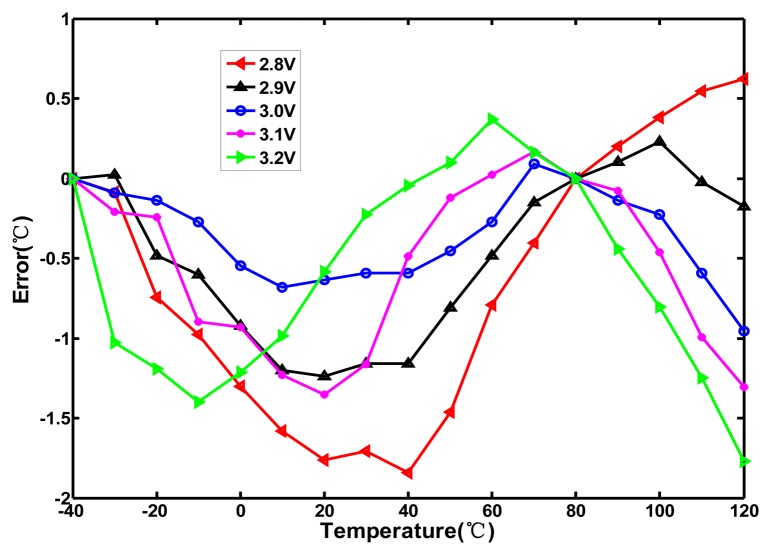
Measurement errors in the range of −40–120 °C for supply voltage variation.

To explore the effect of process variation, all of the effective resolutions of the test chips are calculated to be approximately 0.045 °C; the corresponding histogram is plotted in Figure 12. Without tuning the value of n, the chip-to-chip resolution variation is approximately ±3.3%, which is fairly low for most applications. The linearity of the proposed sensor with voltage sensitivity was also measured from 2.8 to 3.2 V in increments of 0.1 V. Because the compensation oscillator with the voltage variations does not function well, the inaccuracy is degraded to be −1.9–0.7 °C, as depicted in Figure 13. The supply voltage of the proposed sensor should be regulated for reducing the effect of voltage sensitivity. The power consumption was measured as 23 μW at a sample rate of 10 samples/s. With such low power dissipation, it would not cause self-heating to increase the error and any significant increase in the power consumption of the whole system.

## 5. Conclusions

This paper presents an on-chip linearization, time-domain, CMOS smart temperature sensor featuring curvature compensation for accuracy enchantment with a −40 to 120 °C temperature range operation. With the utilization of the curvature compensation oscillator, the linearity was greatly improved without increasing the circuit complexity and chip area. With a supply voltage of 3.0 V, the maximum inaccuracy of 1.4 °C (−1.2–0.2 °C) is achieved after two-point calibration for the 14 test chips. In addition, the temperature resolution can be improved by replacing the two linear delay lines with two ring oscillators. A fine resolution of 0.045 °C is easily realized without significantly increasing the chip area. With such a simple structure and a TSMC 0.35-μm CMOS process, the chip size is extremely small, at 0.07 mm^2^. By taking the CMOS process into consideration, the core area of the proposed sensor is the most favorable among the related smart temperature sensors that ever been reported. The power consumption is measured as 23 μW@10 Hz. These specifications verify that the proposed sensor is suitable for low-power, low-cost, but high-accuracy VLSI integrations. Table 1 summarizes the measured performances of the proposed sensor, as well as those of the related works. Our future work will focus on improving the time-domain curvature compensation technique further to support one-point calibration for reducing the costs of mass production.

## Figures and Tables

**Table 1.** Performances comparison among related time-domain smart temperature sensors.

**Sensor,** **Domain**	**Resolution** **(°C)**	**Error** **(°C)**	**Calibration**	**Reference** **Clock**	**Power** **Consumption**	**Area** **(mm^2^)**	**Temperature** **Range (°C)**	**CMOS** **Technology** **(μm)**
[3], V	0.01	±0.1 (3 σ)	One-point	NA	236 μW@10 Hz	4.5	−55∼125	0.7
[4], V	0.018	±0.25 (3 σ)	One-point	NA	10 μW@10 Hz	0.26	−40∼125	0.16
[5], Time	0.09	±0.6	Two-point	No	9 μW@5 Hz	0.09	−40∼60	0.35
[6], Time	0.058	−1.5∼0.8	Two-point	Yes	8.4 μW@2 Hz	NA	0∼75	0.22/0.18 hybrid
[7], Time	0.3	−1.6∼3.0	Two-point	No	220 nW@100 Hz	0.05	0∼100	0.18
[8], Time	0.3	−0.8∼1	Two-point	Yes	405 nW@1k Hz	0.032	0∼100	0.18
[9], Time	0.139	−5.1∼3.4	One-point	Yes	150 μW@10k Hz	0.01	0∼60	0.065
[10], Time	0.133	−0.7∼0.6	One-point	Yes	175 μW@1k Hz	NA	0∼100	0.22/0.18 hybrid
[11], Time	0.043	−2.7∼2.9	One-point	Yes	400 μW@366k Hz	0.0066	−40∼110	0.065
[12], Time	0.0918	−0.25∼0.35	Two-point	Yes	36.7 μW@10 Hz	0.6	0∼90	0.35
This work, Time	0.045	−1.2∼0.2	Two-point	No	23 μW@10 Hz	0.07	−40∼120	0.35
